# Complete Genome Sequence of Lactobacillus crispatus Type Strain ATCC 33820

**DOI:** 10.1128/MRA.00634-21

**Published:** 2021-08-12

**Authors:** Lucia Teodori, Lorenzo Colombini, Anna Maria Cuppone, Elisa Lazzeri, David Pinzauti, Francesco Santoro, Francesco Iannelli, Gianni Pozzi

**Affiliations:** a Department of Medical Biotechnologies, University of Siena, Siena, Italy; University of Arizona

## Abstract

The complete genome sequence of Lactobacillus crispatus type strain ATCC 33820 was obtained by combining Nanopore and Illumina sequencing technologies. The genome consists of a 2.2-Mb circular chromosome with 2,194 open reading frames and an average GC content of 37.0%.

## ANNOUNCEMENT

**FIG 1:**
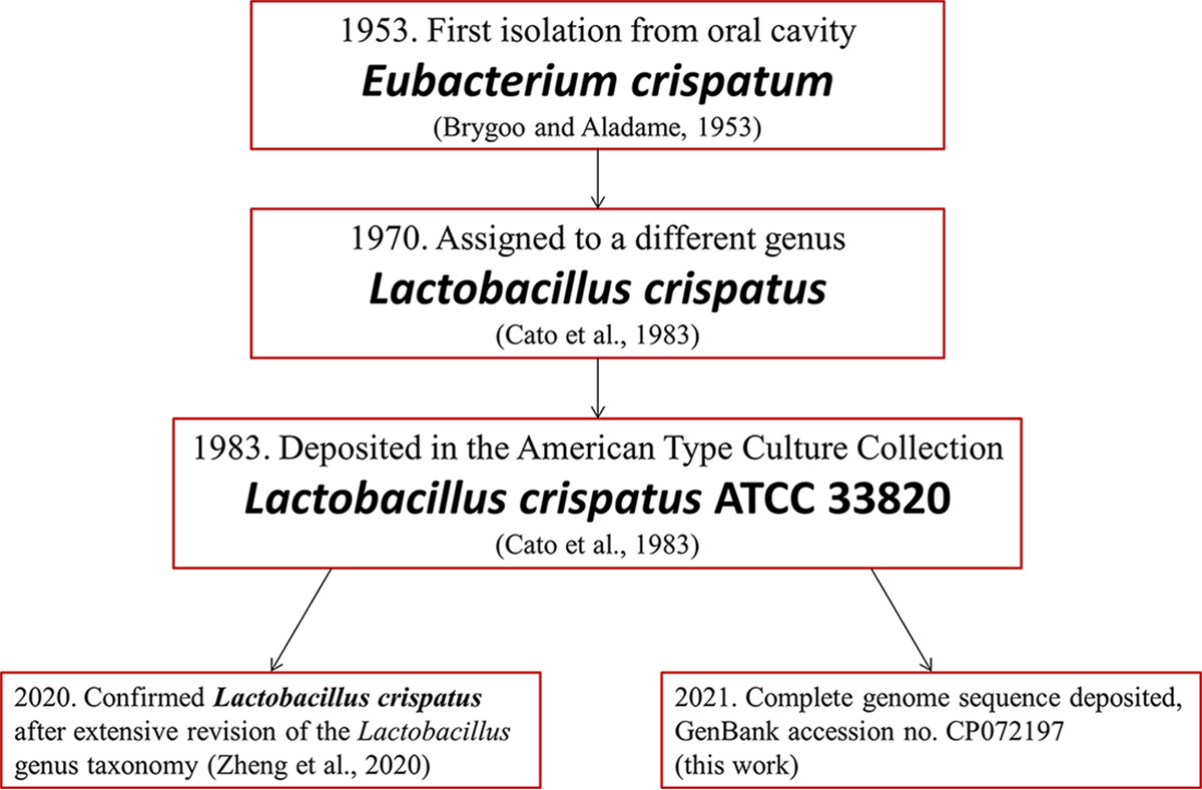
History of Lactobacillus crispatus type strain ATCC 33820. L. crispatus type strain ATCC 33820 was isolated at the Institut Pasteur in 1953 by E. R. Brygoo and N. Aladame from an oral sample of a European individual in Saigon and was considered a new species of the genus *Eubacterium* (Collection of the Institut Pasteur, Paris, strain II) (7). Later, it was deposited in the Virginia Polytechnic Institute and State University as VPI 3199 and identified as *Lactobacillus* (8). Further characterization upon American Type Culture Collection deposition indicated that ATCC 33820 DNA was 100% homologous to the previously defined L. acidophilus group A2 (8). Over the years, the L. crispatus type strain has been distributed among different collections and also designated DSM 20584 = CCUG 30722 = CIP 102990 = CIPP II = JCM 1185 = LMG 9479. Recently, Zheng and colleagues (9) reclassified the genus *Lactobacillus* into 25 genera through a polyphasic approach; however, the nomenclature of Lactobacillus crispatus remained unchanged. Strain ATCC 33820 was acquired by our laboratory in October 2020. Arrows indicate sequential steps in the history of the L. crispatus type strain. Red boxes contain the year, followed by a brief description of the event, the strain name (in bold), and the reference (in parentheses).

Lactobacillus crispatus is the most frequently isolated species among the vaginal lactobacilli of the human microbiota of healthy women; its presence is associated with reduced risk of preterm delivery, viral sexually transmitted infections, and bacterial vaginosis (1). To date (June 2021), only eight L. crispatus complete genomes are available in the NCBI database (https://www.ncbi.nlm.nih.gov/genome/browse#!/prokaryotes/1815/). Here, we contribute to the genomic characterization of this species by publicly releasing the genome of strain ATCC 33820, the type strain of Lactobacillus crispatus (Fig. 1). The strain was purchased from the American Type Culture Collection in October 2020 and grown in 250 ml of DeMan-Rogosa-Sharpe (MRS) broth at 37°C to an optical density at 590 nm (OD_590_) of 1.9. Bacterial cells were harvested by centrifugation (5,000 × *g* for 30 min at 4°C), and the cell pellet was dry-vortexed and incubated for 1 h at 37°C in protoplasting buffer (20% raffinose, 50 mM Tris-HCl [pH 8.0], 5 mM EDTA) containing 4 mg/ml lysozyme. Protoplasts were centrifuged (5,000 × *g* for 5 min), resuspended in 15 ml of deionized H_2_O with 100 μg/ml proteinase K (Merck KGaA, Darmstadt, Germany), and incubated for 30 min at 37°C to obtain osmotic lysis, with 0.5% SDS added after 15 min. Then, 0.55 M NaCl was added, and the mixture was incubated for 10 min at room temperature. High-molecular-weight DNA was purified by three extractions with 1 volume of Sevag (chloroform-isoamyl alcohol, 24:1 [vol:vol]), precipitated in 0.6 volume of cold isopropanol, and spooled on a glass rod. DNA was resuspended in saline-sodium citrate (SSC)/10 buffer and then adjusted to 600 μl SSC 1×. The DNA solution was homogenized using a rotator mixer and stored at 4°C. DNA sequencing was performed with both Oxford Nanopore GridION and Illumina NovaSeq 6000 instruments. The Nanopore sequencing library was prepared using the Nanopore sequencing kit SQK-LSK 109 (Oxford Nanopore Technologies, Oxford, UK), and the sample was sequenced using an R9.4 flow cell (FLO-MIN106). Real-time high-accuracy base calling (quality cutoff, >Q7) of Nanopore reads was performed using Guppy v4.0.11 (https://github.com/nanoporetech/pyguppyclient), and base-called reads were analyzed with NanoPlot v1.18.2 (2). Illumina sequencing was performed at MicrobesNG (University of Birmingham, UK) using a Nextera XT library preparation kit (Illumina Inc., San Diego, CA, USA), followed by paired-end sequencing. Illumina reads were trimmed using Trimmomatic v0.30 (3) and analyzed with FastQC v0.11.5 (http://www.bioinformatics.babraham.ac.uk/projects/fastqc). Nanopore and Illumina sequencing generated 136,000 long reads (630,559,194 bp; *N*_50_, 8.7 kb) and 762,936 read pairs (2 × 250 bp), respectively. Nanopore reads were filtered using Filtlong v0.2.0 with the parameter -target_bases to retain a total of 230 Mbp (https://github.com/rrwick/Filtlong) (*N*_50_, 19,822 bp) and assembled using Unicycler v0.4.7 (4). The resulting circular contig was polished using Medaka v0.7.1 (https://github.com/nanoporetech/medaka) with all Nanopore reads, followed by two polishing rounds with Pilon v1.22 using the Illumina reads (5). Assembly quality was evaluated using Ideel (https://github.com/mw55309/ideel). Annotation was performed with the NCBI Prokaryotic Genome Annotation Pipeline (PGAP) v5.1 (6). Default parameters were used for all software unless otherwise specified. The genome of L. crispatus ATCC 33820 consists of a single circular chromosome (2,239,089 bp) with an overall GC content of 37.0%. The assembly contains 2,194 open reading frames, 78.8% with putative biological function, 64 tRNA genes, 3 rRNA operons, and 3 structural RNAs.

### Data availability.

Sample information and sequence and genomic assembly/annotation are accessible under the NCBI BioProject, BioSample, and whole-genome sequence accession numbers PRJNA716945, SAMN18472633, and CP072197, respectively. Raw Nanopore and Illumina sequencing reads are accessible under Sequence Read Archive accession numbers SRR14509463 and SRR14509462, respectively.
